# Work-life balance in medical students: self-care in a culture of self-sacrifice

**DOI:** 10.1186/s12909-020-02434-5

**Published:** 2021-01-06

**Authors:** Aled Picton

**Affiliations:** College of Medical and Dental Sciences, University of Birmingham, Edgbaston, Birmingham, B15 2TT UK

**Keywords:** Work-life balance, Medical students, Undergraduate, Wellbeing, Self-care

## Abstract

**Background:**

Work-life balance is a key contributor to doctors’ wellbeing and consequently is a central factor in their career decisions. General Medical Council guidance outlines the importance of work-life balance as part of compassionate self-care. Learning self-care should begin as an undergraduate, when academic and clinical workload can contribute to stress, anxiety and burnout.

**Methods:**

Sequential mixed methods study of medical students in Years 3–5 at the University of Birmingham, UK. Students (*n* = 145) defined work-life balance in free-text answers and self-assessed their current work-life balance via questionnaires. Following this, a sub-sample of students (*n* = 44) participated in exploratory individual mini-interviews.

**Results:**

Work-life balance emerged as a broad and multifactorial concept. Questionnaire respondents most frequently referenced enjoyment, meeting work requirements and time management in their definitions. Interview participants highlighted additional influencing factors such as peer groups, study skills, family and professional culture. Students expect a significant shift towards work after graduating and expressed concerns about the stresses of delivering patient care. 42% (*n* = 60) of students felt they had received support with their work-life balance during their training, mostly from family and friends. Most students had not received support or advice on their work-life balance from University or hospital staff.

**Conclusion:**

Self-care and work-life balance are essential for medical students and doctors to cope with lifelong learning and deliver effective care. Medical school staff should be pro-active in supporting students to develop these skills, particularly during critical transition periods. Early interventions targeting study skills and time management may be beneficial. Further research should include students in Years 1–2 and compare different institutions.

**Supplementary Information:**

The online version contains supplementary material available at 10.1186/s12909-020-02434-5.

## Background

Doctors and medical students need to care for themselves as well as their patients, which involves paying close attention to their health and work-life balance [1]. The 2018 General Medical Council (GMC) guidance ‘Outcomes for graduates’ places a stronger emphasis on self-care than previous versions, outlining that *‘newly qualified doctors must demonstrate awareness of the importance of their personal, physical and mental wellbeing and incorporate compassionate self-care into their personal and professional life.’* [2].

Self-care needs to begin at an undergraduate level. Medical students have to balance their time between multiple commitments including teaching, clinical attachments, exams, extra-curricular activities and social life. The combination of academic workload and immersion into a clinical environment can threaten medical students’ work-life balance and wellbeing [3]. International systematic reviews have identified high rates of anxiety, depression and stress amongst medical students [4, 5]. If not addressed, burnout and empathy erosion can follow in some students [5].

An effective work-life balance is hypothesised as a protective factor against these risks when adjusting to university life [6, 7]. It is also a key aspect of self-care when UK medical students transition to the next two stages of their training after graduation: the Foundation Programme and subsequent specialty training.

All UK medical graduates must complete the Foundation Programme. The Foundation Programme is a two-year, work-based training programme which bridges the gap between medical school and specialty training. Foundation Year (FY) doctors provide frontline clinical care to patients and also need to find time for multiple other work-related activities. These include completing a mandatory electronic portfolio of assessments and competencies, alongside participation in teaching and clinical governance activities. Additionally, many doctors study for and sit professional Royal College examinations during their FY training [8].

Due to their rotas and work commitments, UK FY doctors can report finding it difficult or not possible to participate in sports, hobbies and their social lives [9]. Perhaps in an attempt to reset this balance, increasing numbers of doctors are opting to take a career break immediately after the Foundation Programme. In 2018 14.4% of FY doctors took a break at this point compared to 4.6% in 2011 [8, 10]. Motivating factors cited by these individuals such as emigrating or escaping stress could be viewed as self-care strategies or may reflect underlying burnout [8].

Rates of FY2 doctors progressing directly into specialty training have fallen from 71.3% in 2011 to 37.7% in 2018 [8, 11]. These recruitment issues are felt more acutely in certain specialties. Perception of work-life balance and flexibility are key priorities when students and junior doctors choose their postgraduate specialty [12–15]. Both factors are acknowledged to be barriers to recruitment and retention in current UK ‘shortage specialties’ such as Emergency Medicine and Paediatrics [16, 17].

Therefore, the ability to develop a work-life balance and self-care skills may contribute to medical students’ university experience and shape their subsequent career decisions. How students perceive and experience work-life balance merits close examination.

This paper seeks to explore how UK medical students define and manage their work-life balance, their perceptions of work-life balance as qualified doctors and finally the sources of support available to them in achieving a work-life balance.

### Research questions

1. How do medical students define and conceptualise work-life balance?
2. How do medical students perceive their current work-life balance and do they anticipate this changing after graduation?
3. Do medical students receive support with their work-life balance, and if so where do they receive this from?

## Methods

### Study design

Sequential mixed methods study. The study featured a two-stage data collection process: self-completed questionnaires followed by mini-interviews of a sub-sample of students. A pilot study was not carried out.

### Ethical considerations

Ethical approval for the study, carried out as part of a taught Masters programme, was obtained from the University of Birmingham School of Education in March 2016. Participation in both the questionnaire and interview phases was entirely voluntary.

### Setting and population

The study period was March to April 2016. Participants were in the final three years of a five year medical programme (MBChB) at the University of Birmingham, UK. These students spend the majority of their time on clinical attachment. This comprises acquiring basic clinical skills in medical and surgical environments during Year 3 followed by exposure to medical specialties in Year 4. During Year 5, students focus on management of acutely ill patients in preparation for commencing posts as FY doctors.

The study population were all students on placement in a large district general hospital during the study period. This comprised *n* = 82 Year 3 students, *n* = 35 Year 4 students and *n* = 52 Year 5 students. Allocation to hospital placements is random so these students are expected to be widely representative of the overall year cohorts.

### Questionnaire development

At the time of study design, no validated questionnaires of work-life balance in medical students or doctors were identified in the literature. Therefore a novel questionnaire was designed. This was reviewed and refined in discussion with newly qualified doctors (*n* = 4) and the project supervisor (a Senior Lecturer in Medical Education) to check understanding and face validity. The questionnaire comprised four parts and is included in Additional file 1: **Appendix 1**.

Part one of the questionnaire included demographic questions on age, gender, year of study and whether students were on the Graduate Entry Course (GEC). GEC students complete a four year programme: this comprises a standalone first year and students then join the main cohort in Year 3.

Part two focused on how students perceive their work-life balance. It featured a diagram with statements placed at equal intervals between ‘Life’ and ‘Work’ domains as a Likert scale.

Students were then asked the following questions in relation to the diagram:

***Q1:*** *Which of the above five statements (A-E) best summarises your own assessment of your* ***current*** *work-life balance,* ***based on this academic year as a whole?***

***Q2:*** *Which of the above five statements (A-E) best summarises where you think your work-life balance should be* ***at your current stage of training****?*

***Q3:*** *Which of the above statements (A-E) best summarises how you anticipate your work-life balance may be* ***as a Foundation Year doctor?***

Part three featured an open-ended question: *‘what does work-life balance mean to you?’* to elicit student perceptions. Finally, part four asked students if they had received any support or training on work-life balance, and if so from what source. Students were then asked what could help them to improve their work-life balance. Questions requiring free-text responses were placed at the end of the questionnaire. The rationale for this was to enable students to acclimatise to the topic prior to their completion.

### Data collection

All students on placement at the hospital trust were contacted via email to inform them of the study prior to data collection commencing. Questionnaires were distributed during teaching sessions and made available for students who did not attend. After completing the questionnaire, students were asked whether they would be willing to complete an individual interview with the researcher to discuss the topic of work-life balance. If a student volunteered for an interview, they were contacted by the researcher and an interview arranged.

### Interview methodology

Exploratory individual mini-interviews with a sub-sample of students took place after the questionnaire phase, to provide students with an opportunity to expand on their responses. Interviews featured eleven questions with optional prompt questions- these are included in Additional file 2: **Appendix 2**. The questions included:

- *How do you switch off?*
- *Is there anything that currently helps you to achieve a work-life balance?*
- *Do you anticipate changes to your work-life balance in the future?*
- *What differences (if any) do you think there are to a work-life balance in medicine, compared to other careers?*

Interviews took place during work hours on hospital premises. Interview length ranged from 6 to 19 min.

#### Data handling and analysis

Interviews were recorded using a voice recording smartphone app (Voice Record Pro, Dayana Networks Ltd) then transcribed by the researcher. Transcripts were read, re-read and then thematically analysed [18]. Qualitative data from interviews and questionnaires were managed and coded using NVivo (Version 11.1.0, QSR International®).

Quantitative data were inputted into SPSS Statistics (Version 24, IBM®). Non-parametric analysis was carried out across the three year groups for each question via one-way ANOVA testing. Graphs and results were generated in SPSS Statistics and Microsoft Excel (Version 15.23.2, Microsoft®).

## Results

### Study participants

**Fig. 1:**
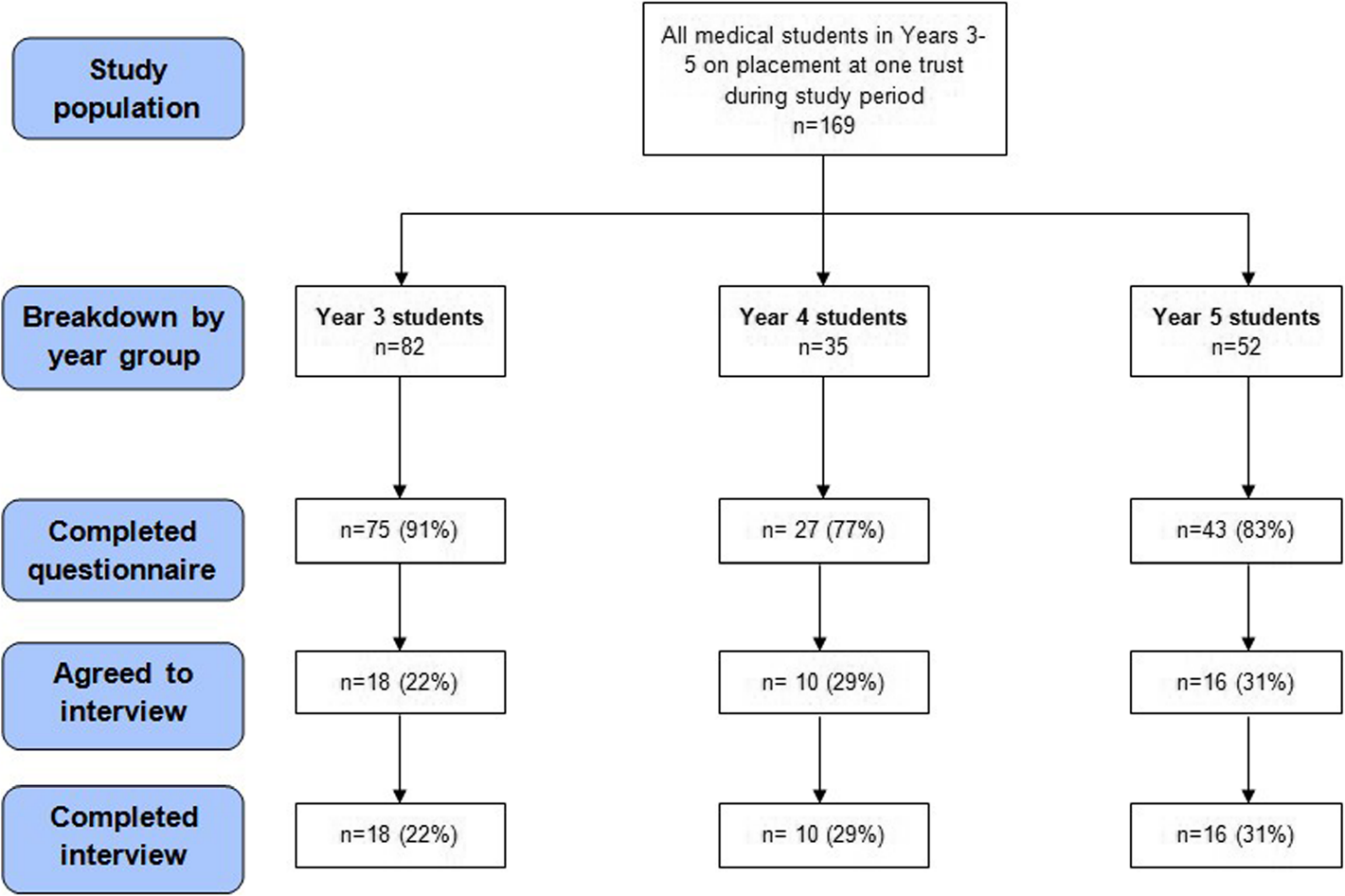
Study flow chart

Figure 1 shows questionnaire respondents and interview participants. Both groups were representative of the overall cohort in regard to gender (65% female). Table 1 outlines participant characteristics which are briefly summarised here:

**Table 1:** Participant characteristics

		Year 3	Year 4	Year 5	All years
**Questionnaire** respondents					
**Gender**	M	26 (34.7%)	8 (29.6%)	15 (34.9%)	49 (33.1%)
	F	49 (65.3%)	19 (70.4%)	28 (65.1%)	96 (66.9%)
**Age range (years)**		20–31	21–27	22–27	20–31
**GEC**		4 (5.3%)	5 (18.5%)	5 (11.6%)	14 (9.7%)
**Interview** participants					
**Gender**	M	4 (22.2%)	2 (20%)	7 (43.8%)	13 (29.6%)
	F	14 (77.8%)	8 (80%)	9 (56.2%)	31 (70.4%)
**Age range (years)**		20–23	21–24	22–27	20–27
**GEC**		0	2 (20%)	4 (25%)	6 (13.6%)

***A: Questionnaire respondents.***

There were *n* = 145 questionnaire respondents (response rate 86%) of which two thirds were female (*n* = 96, 67%). Age range of respondents was 20–31 years.

***B: Interview participants.***

Approximately one third of questionnaire respondents agreed to interview and *n* = 44 individual interviews were recorded. Interview participants were *n* = 18 Year 3 students, *n* = 10 Year 4 students and *n* = 16 Year 5 students. 70% of participants were female (*n* = 31). Age range was 20–27 years.

#### **1. How do students define and conceptualise work-life balance?**

Questionnaire respondents wrote a mean of 23 words for their free-text definitions, range 0–78 words. Mean answer lengths in words were similar when compared by year group and gender. Core themes from questionnaire data were enjoyment, meeting work requirements and time management, as evidenced by this student’s definition of work-life balance:

*‘The ability to balance work commitments with personal time in which you are able to relax, socialise and pursue extra-curricular interests.’* (Year 3 student).

**Fig. 2:**
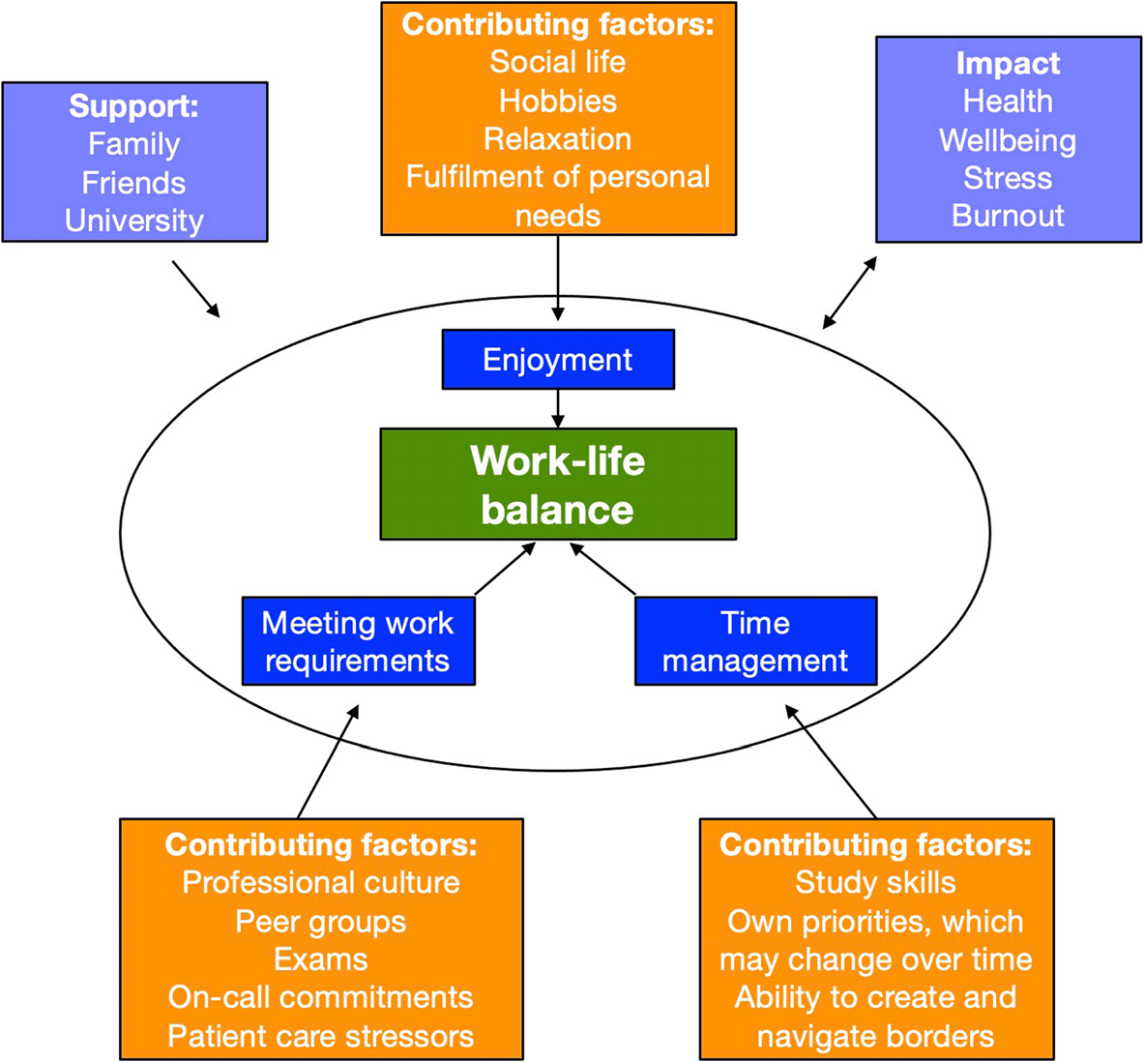
Synthesis of questionnaire and interview data

These core themes recurred in interviews. Analysis of interview data identified additional contributing factors to work-life balance such as peer groups, study skills, professional expectations and family. Figure 2 synthesises and triangulates both questionnaire and interview data. Enjoyment, meeting work requirements and time management are located at the centre. These are intrinsic factors governed by an individual’s work ethic and priorities.

## Questionnaire data

### Enjoyment

Enjoyment was the most frequently referenced theme from questionnaire data and was included by over two thirds of respondents, for example:

*‘Prioritising life: should always find time to do things that you enjoy.’* (Year 3 student).

Questionnaire respondents brought up a diverse range of individual and social pursuits, with hobbies, team sports and exercise frequently referenced. Students often referenced their social lives, emphasising friends, families and partners. For example:

*‘To make the most of other aspects of life such as time for meeting up with friends and maintaining relationships.’* (Year 5 student).

### Attitudes to work

Questionnaire respondents employed two different approaches when describing their work. The more common option, ‘meeting work requirements’ encompassed students who described doing the minimum or satisfactory amount of work and completing this on time- for example:

*‘I complete what is expected of me.’* (Year 3 student).

In contrast a minority of respondents put more emphasis on work. These students outlined commitment, enthusiasm or aspiration within their studies and career. For example:

*‘Having a satisfying job which you find rewarding and interesting.’* (Year 5 student).

*‘Give 100% to the job.’* (Year 3 student).

### Time management

Many questionnaire respondents acknowledged in their definitions that achieving a work-life balance is an active process. Time management was a recurring theme, which involved:

*‘Organising yourself ahead of time.’* (Year 4 student) *‘Devoting enough time and energy.’* (Year 5 student).

### Cynicism

A small group of questionnaire respondents (*n* = 3) across Years 3 and 4 defined work-life balance with a degree of cynicism or futility:

‘*It is not really achievable.’* (Year 3 student).

*‘A false concept’.* (Year 4 student).

As questionnaires were anonymous it was not possible to identify if these particular students participated in interviews.

## Interview data

Interview data provided a more in-depth insight into students’ perceptions of work-life balance. Interview participants had contrasting thoughts on how pro-actively they monitored their work-life balance. Some students emphasised the importance of continual self-assessment in this area, for example:

*‘I just try to make a conscious effort and think, you know what- work is always going to be there, I need to stop and do something I like.’* (Year 3 student).

In contrast, some students felt the opposite way:

*‘I never think about the balance … I never think I’m working too much or that I haven’t done anything that’s not related to work in a while. That’s probably quite bad.’* (Year 3 student).

### Peer groups

Peer groups were the most commonly coded aspect of interview data. Interview participants described an immersive undergraduate experience wherein peer groups, living arrangements and a competitive culture between students shaped their work-life balance. Students’ term-time living arrangements and work ethic of peers could motivate but could also lead to guilt and anxiety, as evidenced by these contrasting quotes:

‘*Last year I lived in quite a work-orientated household so I definitely did more work because of that.’* (Year 3 student).

‘*Doing a medical degree I do feel guilty when I’m not working and I know other people are working.’* (Year 5 student).

### Learning study skills

Interview participants reflected on how they had developed their self-directed learning skills over the course of the degree. Year 3 students in particular discussed the challenging transition from an achievable syllabus in sixth form or college to studying medicine. For example:

*‘In first year I was all over the place, I couldn’t figure it out. Wanted to make friends, wanted to go out. But I also had all this work to do, but didn’t know how to do it because it’s so different to school when you’re spoon-fed, given all the information.’* (Year 3 student).

Students in all years acknowledged that medicine is an infinite topic, with a requirement for lifelong learning. This was highlighted as a source of stress and anxiety, for example:

‘*There is no good end point with medicine. There is always something more you could be doing.’* (Year 3 student).

Interview participants reflected on how they adjusted their working strategy with experience, often based on trial-and-error, to become more effective in their study skills. One student commented:

*‘Because I’ve been at medical school, especially a clinical student for quite a few years now, I’ve learned what to prioritise. How to get the most from my learning. I think medicine is very much about working smart as well as working hard’.* (Year 5 student).

### Creating borders

A minority of interview participants, particularly in Years 4 and 5, discussed how they created clear borders to help achieve a work-life balance. For example:

*‘I dedicate my weekdays to my working life. My weekends I just like to completely switch off.’* (Year 5 student).

These students generally expressed a motivating factor, for example the working hours of a partner or previous experiential knowledge such as full-time employment. Some used time boundaries to separate their life and work domains. Others discussed using different environments, for example choosing to exclusively work in the library rather than at home.

### Stress and burnout

Interview participants acknowledged stress, anxiety and burnout as potential consequences of a poorly-maintained work-life balance. Interviewees felt that to maintain a work-life balance, both work and life aspects should ideally have minimal impact on each other. For example:

*‘Time for relaxing that does not result in building up of responsibility or neglect of professional values.’* (Year 3 student).

Interview participants acknowledged that this could work both ways, and over-committing to work could have detrimental consequences for themselves and potentially patients. This was particularly highlighted by Year 5 students, for example:

*‘Without letting work dominate your life or burning out.’* (Year 5 student).

The majority of students discussed stress and burnout in an abstract sense and some talked about peers who had first-hand experience. A significant degree of workload variability across each academic year, largely due to exams, was emphasised across all year groups. A few interview participants described feeling exhausted or overwhelmed due to their current workload, for example:

*‘Your mindset is always thinking about work. You can’t get away from it. You go home and it’s still there. You don’t get to eat, don’t get to sleep.’* (Year 4 student).

### Culture and expectations within medicine

Interview participants discussed a professional culture of self-sacrifice within medicine and expressed concerns about maintaining a work-life balance, for example:

*‘I would like to be a well-rounded person and I can see myself getting funnelled into a medicine lifestyle where you are living and breathing it.’* (Year 5 student).

Interview participants in Years 4 and 5 had a growing appreciation of the responsibility and potential stresses of delivering patient care. Some were concerned about not being able to ‘switch off’ at home after work. For example:

*‘In medicine there is potential … for bringing work home with you. Worry about mistakes that you’ve made, worrying about someone on death’s door- you left, will they be alright?’* (Year 4 student).

### Family, upbringing and priorities

Interview participants compared studying medicine and its associated expectations with the experiences of family members in other professions. For example:

*‘In medicine you have to do a lot of studying. You tend to take your work home with you. Whereas my dad is a hairdresser- once he’s finished cutting hair he comes home and that’s the end of the day.’* (Year 4 student).

Interviewees also reported that parents’ working patterns could influence their own expectations of work-life balance:

*‘If your parents worked all the time, or stayed at home, you would have an idea of what work and life should be like.’* (Year 4 student).

Interview participants in Year 5 described how they and their peers experienced changes to their lives and priorities over the course of the degree. Life events such as getting married or having children affected their perspectives on work-life balance. For example:

*‘Medicine just doesn’t seem to be everything any more. There is so much more going on in life. People are getting married, having kids.’* (Year 5 student)

#### 2. How do medical students perceive their current work-life balance and do they anticipate this changing after graduation?

Questionnaire respondents had broadly similar expectations of what their work-life balance ‘should be’ in each year, typically between *an equal balance of work and life* and *a partial shift towards work*. Students assessed their actual work-life balance as similar to what they thought it ‘should be’, with the exception of Year 5 who reported their current work-life balance as closer towards *an equal balance* than they had expected.

**Fig. 3:**
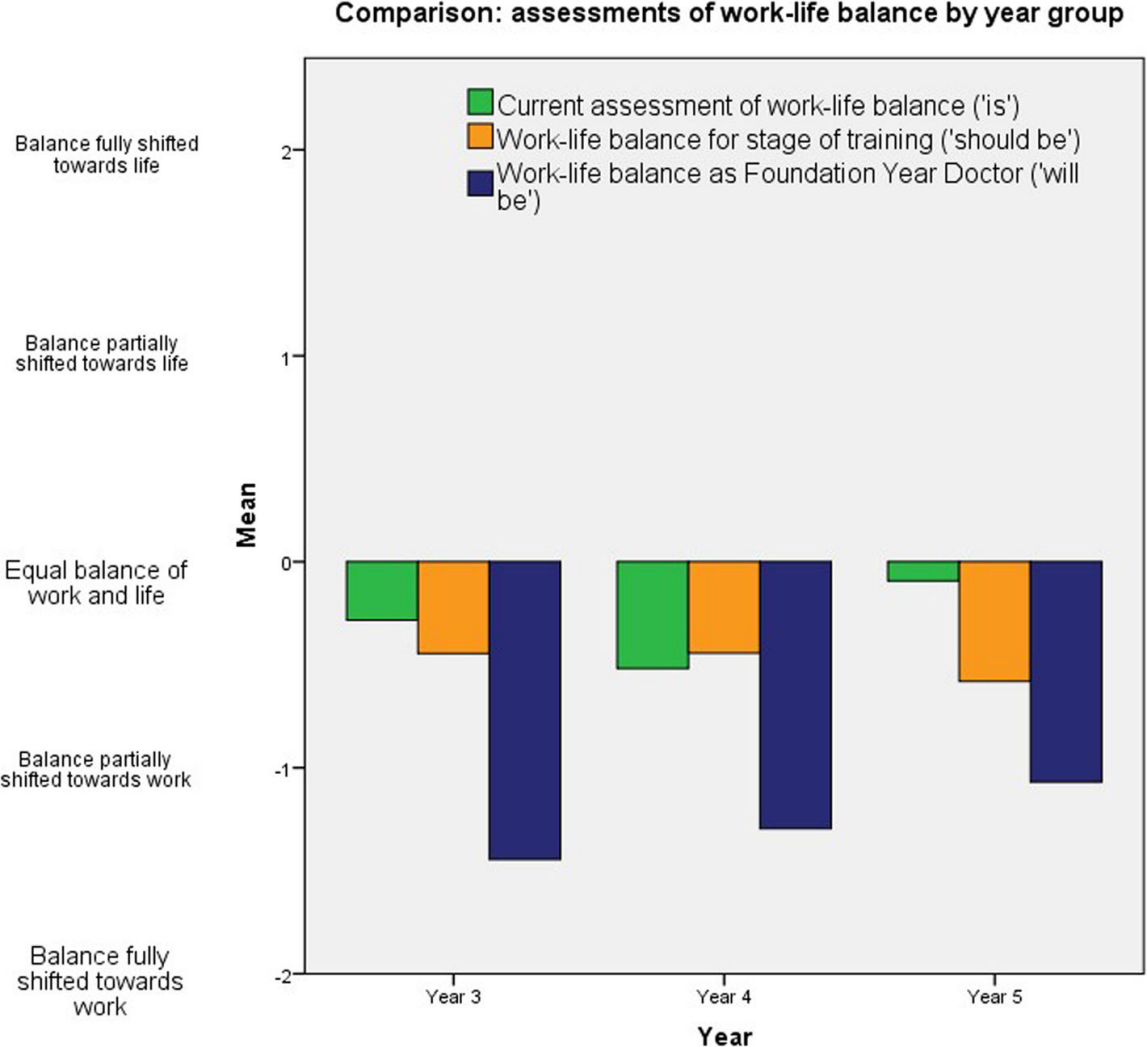
Graph comparing assessments of work-life balance by year group

Looking ahead to FY training, questionnaire respondents anticipated a shift towards work compared to their current work-life balance. However year groups had different expectations, with students in Year 3 anticipating a greater emphasis on work during FY training than students in Year 5. There were no statistically significant differences between genders for any of these questions. See Table 2 and Figure 3 for questionnaire results.

**Table 2:** Questionnaire results

			Year 3		Year 4		Year 5		All years	
			n=	%	n=	%	n=	%	n=	%
**Current work-life balance**	Full shift to work	A	3	4.1	3	11.1	2	4.7	8	5.6
	Partial shift to work	B	36	48.6	11	40.7	17	39.5	64	44.4
	Equal balance	C	15	20.3	10	37	7	17.6	33	22.9
	Partial shift to life	D	19	25.7	3	11.1	15	34.9	37	25.7
	Full shift to life	E	1	1.4	0	0	1	2.3	2	1.4
**Stage of training expectations**	Full shift to work	A	6	8	2	7.4	1	2.3	9	6.2
	Partial shift to work	B	25	33.3	11	40.7	25	58.1	61	42.1
	Equal balance	C	40	53.3	11	40.7	15	34.9	66	45.6
	Partial shift to life	D	4	5.3	3	11.1	1	2.3	8	5.5
	Full shift to life	E	0	0	0	0	1	2.3	1	0.7
**FY training expectations**	Full shift to work	A	36	48	10	37	16	37.2	62	42.8
	Partial shift to work	B	35	46.7	15	55.6	17	39.5	67	42.6
	Equal balance	C	4	5.3	2	7.4	7	16.3	13	8.9
	Partial shift to life	D	0	0	0	0	2	4.7	2	1.4
	Full shift to life	E	0	0	0	0	1	2.3	1	0.7
**Support with work-life balance**	Yes		38	50.7	10	38.5	12	27.9	60	41.6
	No		19	25.3	9	34.6	24	55.8	52	36.1
	Not sure		18	24	7	26.9	7	16.3	32	22.2

Interview participants anticipated a different work-life balance after graduating. Students expected that during the Foundation Programme, on-call commitments may limit their amount of rest, socialising and extra-curricular activities. For example:

*‘It’s quite scary. On-calls, weekend shifts. That’s definitely going to change how we can go on with a routine, keeping up with friends and family. I do anticipate a change.’* (Year 4 student).

*‘In the back of my mind, there’s probably this idea that this is just temporary- getting rid of your hobbies, putting everything on hold. But I wonder if perhaps after this it’s only going to get worse.’* (Year 5 student).

However in contrast to this, some students in Year 5 were able to highlight positive aspects of postgraduate life, for example earning a salary, which meant they felt their work-life balance may improve after graduation:

*‘I think there’s going to be a bit more work to do, but on the other hand a bit more financial freedom to do things that will benefit the life aspect of the work-life balance. Simple things like being able to go and have a nice meal out without having to worry about money or booking a holiday.’* (Year 5 student).

####  **3. Do medical students currently receive support with their work-life balance, and if so where do they receive this from?**

Across all year groups, 41.6% of questionnaire respondents (*n* = 60) felt they had received guidance, support or advice with their work-life balance during their studies. One third (36.1%, *n* = 52) felt they had not and 22.2% (*n* = 32) were not sure. There were differences present between year groups, with students in Year 5 least likely to report receiving support.

These findings were also present in interview data. Some interview participants felt that they had received minimal support or input from university staff on work-life balance, particularly in their early years of training and voiced regret or frustration about this:

*‘I wish I had known from the outset I could have a work-life balance.’*

(Year 5 student)

For those that reported receiving support, the most common sources cited were family (30.5% of all students, *n* = 44) and friends (26.3%, *n* = 38). Personal mentors were the most common source of support provided by the medical school acknowledged by students (22.2%, *n* = 32), followed by teaching (11.8%, *n* = 17), Senior Academy Tutors (6.9%, *n* = 10) and welfare staff (5.6%, *n* = 8). Senior Academy Tutors are doctors who carry out formal teaching for students during their clinical attachments.

## Discussion

### Summary of findings

Enjoyment, meeting work requirements and time management were recognised as key intrinsic contributors to work-life balance in questionnaire data. Leisure time is important as it provides students and doctors with an opportunity to fulfil personal needs, rather than satisfying the needs of others. A strong emphasis on enjoyment in students’ work-life balance definitions supports this.

Individual interviews allowed students to express their thoughts and perceptions on this topic in more detail, and other factors arose more prominently. These included how peer groups, study skills, family and professional culture can all influence work-life balance.

Students anticipated a shift towards work on starting FY training, but expectations for a future work-life balance were much further towards work for Year 3 students than for Year 5 students. It is possible that for Year 3 students who had recently made the challenging transition to full time clinical attachments, the proposition of their next major step up appeared particularly daunting. Conversely, Year 5 students anticipated a less work-heavy balance in FY training and highlighted perceived benefits of postgraduate life such as increased financial freedom. These findings may be due to their proximity to this change, an enhanced role understanding through shadowing and discussion with peers already undertaking FY training.

Less than half of all students (41.6%, *n* = 60) felt that they had received support or guidance with their work-life balance. Family and friends were the most commonly cited sources of help. Students in Year 5 were the least likely to report receiving support (27.9%, *n* = 12), despite this being a challenging year of study and students’ proximity to starting the Foundation Programme. It is unclear why students in Years 4 and 5 were less likely to report receiving support than those in Year 3. These differences should be interpreted cautiously given the different numbers of questionnaire respondents in each year group.

#### Self-care or self-sacrifice?

Self-care and altruism can be viewed as aspects of professionalism that sit along a continuum. GMC guidance and medical education literature has recently shifted towards self-care but prior to this, the preceding ethos emphasised altruism and self-sacrifice [19].

Interview participants discussed an underlying professional culture of self-sacrifice within medicine that could influence their work-life balance. Students felt that this was cross-generational and influenced medical students as well as practising doctors. These perspectives on medical culture and work-life balance are concerning and suggest notions of self-sacrifice may still permeate some aspects of medical education and practice, perhaps via the hidden curriculum.

Overall, students had variable awareness and insight into their work-life balance. Some interview participants reflected that participating in the study process had led them to consider this for the first time. Others acknowledged that they found it difficult or chose not to reflect on their work-life balance. Interview studies with small numbers of UK FY doctors highlight that many only apply this concept to themselves after graduating [9]. This appears to be mirrored in the study findings.

#### Navigating borders

It is hypothesised in occupational literature that to achieve work-life balance, professionals need to be able to manage and negotiate work and family spheres, including the borders between them [20]. Borders can be interpreted as geographical, chronological or psychological ways of separating work and home environments. Border theory was proposed for working professionals rather than university students. Only a minority of students employed this approach, and these students either had previous experience of full-time employment or a partner who worked full-time. Border theory may become relevant to more students when they begin the Foundation Programme.

In contrast to clear borders, the majority of interviewed students described a university experience which featured blurred boundaries between work and personal life. For example, a lot of their self-directed learning took place in their home environment, often with fellow medical students. This offered the benefits of social learning, but also drawbacks if students found it difficult to switch off and relax when others were working.

#### Burnout

It is hypothesised that the sustained challenges of medical school can lead to burnout and erosion of empathy in some students, particularly in the later stages of the course [5]. Several students in this study described experiencing stress, anxiety and feeling overwhelmed in either free-text questionnaire responses or during interviews. These perspectives may in part be due to variability across the academic year and data collection occurring close to high-stakes end of year exams. However, they may also hint at undetected cases of burnout amongst some of the study cohort.

#### Gender preferences

Male and female medical students can have different perceptions and expectations of work-life balance, particularly when anticipating their future careers [12]. In contrast to previous research, male and female medical students in this study gave similar responses throughout with no significant differences between group means identified for any question. Gender differences may become apparent when students begin their postgraduate careers, as factors such as working patterns and balancing family commitments are likely to play a prominent role for more individuals at this stage [21, 22].

#### Family and priorities

In keeping with previous research, students in Year 5 described changes to their lives and priorities over the course of the degree which affected their perspectives on work-life balance [12].

The observation that parental role modelling can shape students’ expectations of work-life balance is a new finding in this setting. This highlights that as widening participation schemes develop and the student body becomes more heterogeneous, students entering medical school from ‘non-traditional’ backgrounds (for example, first in family to attend university) may provide different perspectives to their peers.

#### Recommendations for educational practice

Medical students juggle multiple commitments and many express stress or uncertainty about their current and future work-life balance. To support students in maintaining their health and wellbeing during the challenges of a medical degree, teaching staff should reflect GMC guidance and emphasise self-care. This requires targeted interventions at different times.

Students emphasised that learning study skills and time management at the outset of their degree programme was an important step in formulating an effective work-life balance. This was a particular challenge for students who started undergraduate medicine directly from a school or sixth form learning environment. Staff should aim to support this process, for example via study skills workshops or drop-in sessions.

Medical students in Years 4–5 may benefit from additional support or guidance with their work-life balance, given that they encounter different challenges relating to patient care stressors and proximity to starting the Foundation Programme. This could be via existing support mechanisms such as academic tutors. Inviting the involvement of current FY doctors could ensure this content was relevant, as students currently acknowledge these near-peers as an informal source of advice.

As all medical students experience significant workload variability during an academic year, staff should be particularly vigilant regarding self-care messages before and during assessment periods.

### Strengths and weaknesses of study

Strengths of the study include a mixed methods approach, new research questions and high questionnaire response rates. Limitations are acknowledged. This study was designed as an exploratory, pragmatic assessment of students’ work-life balance rather than a full psychometric evaluation. The novel questionnaire was reviewed by a Senior Lecturer in Medical Education and four newly qualified doctors, but was not reviewed by medical students and did not undergo any further assessment.

All participants were from one institution, the University of Birmingham. This may limit generalisability of results to other settings. Students’ perceptions of work-life balance may have been affected by institution-specific factors. These factors could be explicit or transmitted via the hidden curriculum.

Recruitment of participants who were all on placement at one hospital trust was an opportunistic and pragmatic approach to sampling. A sample size calculation was not carried out prior to recruitment of participants. Numbers of respondents per year group were different for the questionnaire phase (*n* = 75 Year 3, *n* = 27 Year 4, *n* = 43 Year 5) and may limit conclusions drawn.

Data were collected approximately one to two months before students’ end of year exams. Fatigue, stress and different working patterns may have influenced students’ answers. The sequential design meant that interviews took place closer to exams than the questionnaire phase. This may explain why comments from a minority of interviewed students about feeling overwhelmed or exhausted are not reflected in the questionnaire results.

Triangulation is evidenced through use of multiple methods of data capture, plus comparison of participants’ accounts with existing theoretical schemes such as border theory. However investigator triangulation is absent as the qualitative analysis was carried out by one researcher. This is a limitation of the study.

Both questionnaires and interviews were voluntary so responder bias may affect generalisability of results. Participating students may have been more stressed and looking for an outlet to discuss this or conversely less stressed as they had time available to participate. Students in Years 1–2 were not included and may have provided a different perspective.

## Conclusion

Self-care and work-life balance are essential for medical students and doctors to cope with lifelong learning and deliver effective care. Work-life balance is a broad and multi-factorial concept centred on an intrinsic balance between meeting work requirements and enjoyment, achieved via time management skills. It is shaped by extrinsic factors such as peer groups, family and professional culture. Students anticipate a significant shift from their current work-life balance when they start the Foundation Programme and expressed concerns about the implications of having increased responsibility for patients. Most students did not receive support or advice on their work-life balance from University or hospital staff during their training. To support students in maintaining their health and wellbeing during the challenges of a medical degree, teaching staff should reflect GMC guidance and emphasise self-care, particularly during critical transition periods.

### Practice points

- Self-care is essential for medical students and doctors
- Medical students perceive work-life balance as a broad and multi-factorial concept influenced by peers, family and professional culture
- Self-directed learning and time management skills are key to development of an effective work-life balance
- Less than half of students reported receiving support with their work-life balance
- Future research should include students in Years 1–5 and across different institutions

## Supplementary Information

**Additional file 1.** Appendix 1 Questionnaire. Study questionnaire. Questionnaire used in study.

**Additional file 2.** Appendix 2 interview questions and prompts. Interview questions and prompts. Questions and prompts used in study interviews.

## Data Availability

The datasets used and/or analysed during the current study are available from the corresponding author on reasonable request.

## Abbreviations

FY: Foundation Year
GEC: Graduate Entry Course
GMC: General Medical Council
UK: United Kingdom
