# Supplementary material for: Work-life balance in medical students: self-care in a culture of self-sacrifice
Source: BMC Med Educ. 2021 Jan 6;21:8. doi: 10.1186/s12909-020-02434-5 (PMC7786898; doi:10.1186/s12909-020-02434-5)
Supplement: Supplementary file 1 — Additional file 1. Appendix 1 Questionnaire. Study questionnaire. Questionnaire used in study. [file 12909_2020_2434_MOESM1_ESM.pdf]

UNIVERSITY OF BIRMINGHAM

Work-life balance

What does this mean to medical students at different stages of training, and are they achieving it?

Thank you for reading the study information sheet. If you are happy to continue, please complete the following questionnaire. For each question with options, **please tick the box** indicating your response.

Part 1: **Demographics**

1. What is your gender? ☐ Male ☐ Female

2. What is your age?

3. Are you a Graduate Entry Course (GEC) student?

   ☐ Yes ☐ No

4. What is your current year of study?

Year 3 ☐ (Year 2 GEC)

Year 4 ☐ (Year 3 GEC)

Year 5 ☐ (Year 4 GEC)

UNIVERSITY OF BIRMINGHAM

Student questionnaire

Part 2: **Assessment of work-life balance**

The following diagram forms a simple representation of work-life balance to provide a visual aid for the questions below.

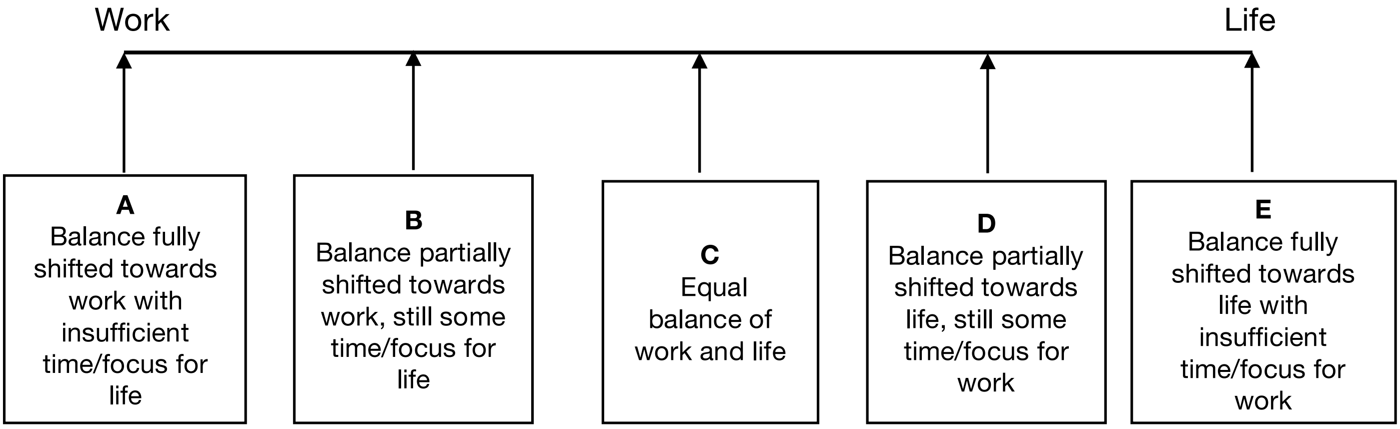

Which of the above five statements (A-E) best summarises your own assessment of your **current** work-life balance, **based on this academic year as a whole?**

A ☐ B ☐ C ☐ D ☐ ☐ E

Please explain why you chose this option:

Which of the above five statements (A-E) best summarises where you think your work-life balance should be **at your current stage of training**?

A ☐ B ☐ C ☐ D ☐ ☐ E

Please explain why you chose this option:

Which of the above statements (A-E) best summarises how you anticipate your work-life balance may be **as Foundation Year doctor?**

A ☐ B ☐ C ☐ D ☐ ☐ E

Please explain why you chose this option:

Part 3: **What does 'work-life balance' mean to you?**

Part 4: **Support and advice**

Have you received any guidance, support or advice on work-life balance during your studies on this course?

☐ Yes ☐ Not sure ☐ No

If you answered yes, which of these best describes where this guidance, support or advice came from. Please tick all that apply:

Formal teaching ☐

Senior Academy Tutor ☐

Personal mentor ☐

Welfare tutor ☐

Family ☐

Friends ☐

Other (please specify below) ☐

What could help you to improve your work-life balance?
