# Supplementary material for: Work-life balance in medical students: self-care in a culture of self-sacrifice
Source: BMC Med Educ. 2021 Jan 6;21:8. doi: 10.1186/s12909-020-02434-5 (PMC7786898; doi:10.1186/s12909-020-02434-5)
Supplement: Supplementary file 2 — Additional file 2. Appendix 2 interview questions and prompts. Interview questions and prompts. Questions and prompts used in study interviews. [file 12909_2020_2434_MOESM2_ESM.docx]

**Appendix 2:** Interview questions and prompts

1. What does work-life balance mean to you?
   *Prompt: Can you tell me a bit more about that?*
2. How do you switch off?
   *Prompt: For example, how do you relax or clear your mind of work activities?*
3. How does an individual’s ‘mindset’ affect how they perceive their work-life balance?
   *Prompt: For example- two people with the same work and life commitments may perceive this balance very differently because of individual factors. Why do you think this might be?*
4. What is good about your current work-life balance?
5. What is bad about your current work-life balance?
6. Do you think there are any other factors related to work-life balance that might affect students on this course?
   *Prompt: For example, can you think of anything that might affect other students with different work or life circumstances?*
7. Is there anything that currently helps you to achieve your work-life balance?
8. Is there anything that currently hinders you in achieving your work-life balance?
9. Do you feel that your work-life balance has changed as you have progressed through medical school?
   *Prompt: Why do you think this is? Have your work or life commitments changed? Have you changed?*
10. Do you anticipate changes to your work-life balance in the future?
    *Prompt: Can you tell me a bit more about this?*
11. What differences do you think there are to a work-life balance in medicine, compared to other careers?
    *Prompt: Do you think this is related to the hours worked, the nature of the work or both?*
